# Biomarker selection and reporting architecture in human proximal tubular *in vitro* models of cisplatin-induced nephrotoxicity: a systematic review

**DOI:** 10.3389/ftox.2026.1862941

**Published:** 2026-07-02

**Authors:** María Gabriella Rodríguez-Marcano, Ornella Pierucci, Jesús Romero Lugo

**Affiliations:** 1 Faculty of Medicine, Universidad de Las Américas, Quito, Ecuador; 2 Health Sciences Faculty, School of Medicine, Universidad Internacional SEK (UISEK), Quito, Ecuador

**Keywords:** acute kidney injury, biomarkers, cisplatin, in vitro models, nephrotoxicity, proximal tubule

## Abstract

Cisplatin-induced acute kidney injury remains a major dose-limiting complication in oncology. Human proximal tubule *in vitro* models are widely used to investigate nephrotoxic mechanisms; however, biomarker selection and reporting patterns vary considerably across studies. This PRISMA-guided systematic review aimed to characterize the frequency, biological categorization, and co-reporting patterns of biomarkers used in human proximal tubule *in vitro* models of cisplatin-induced injury. A systematic search of PubMed, Scopus, and Web of Science identified experimental *in vitro* studies published between 2014 and 2024 that reported quantifiable biomarkers following cisplatin exposure. Biomarkers were classified into predefined structural injury, regulated cell death, and mechanistic categories. Descriptive frequency analyses were performed, and co-occurrence relationships among the 12 most frequently reported biomarkers were evaluated using a binary presence–absence matrix. Fifty-eight studies met inclusion criteria. Kidney injury molecule-1 was the most frequently reported structural biomarker. Mechanistic endpoints, particularly oxidative stress and intracellular signaling markers, predominated over structural injury markers. Co-reporting analysis revealed recurrent pathway-oriented groupings, suggesting hypothesis-driven panel selection rather than standardized implementation across studies. These findings highlight the need for improved harmonization and structured biomarker strategies to enhance reproducibility, predictive robustness, and translational alignment in vitro nephrotoxicity research.

## Introduction

Cisplatin is a platinum-based chemotherapeutic agent widely used in the treatment of solid tumors; however, its clinical effectiveness is frequently limited by dose-dependent nephrotoxicity. The proximal tubule is particularly susceptible to cisplatin accumulation due to active uptake mechanisms and limited detoxification capacity, making cisplatin-induced acute kidney injury (AKI) a clinically relevant adverse effect (Pabla and Dong, 2008; Yao et al., 2007; Miller et al., 2010).

At the cellular level, cisplatin nephrotoxicity involves a complex interplay of oxidative stress, mitochondrial dysfunction, inflammatory signaling, and regulated cell death pathways. Excessive reactive oxygen species generation and mitochondrial impairment are central features of tubular injury and are commonly associated with apoptosis and other forms of regulated cell death (Santos et al., 2007; Miller et al., 2010). These interconnected mechanisms have led investigators to employ diverse structural injury markers, mechanistic endpoints, and cell death–associated biomarkers in experimental systems.

Human proximal tubular *in vitro* models—including immortalized cell lines such as HK-2 and RPTEC/TERT1, as well as organoid and microphysiological platforms—have become widely used tools for mechanistic investigation and nephrotoxicity assessment (Faria et al., 2019; Jang et al., 2013; Homan et al., 2019; Simon-Friedt et al., 2015). Within these systems, a broad array of structural, functional, and molecular endpoints is used to characterize injury responses, often without standardized reporting frameworks. Structural injury biomarkers such as kidney injury molecule-1 (KIM-1) and neutrophil gelatinase-associated lipocalin (NGAL) are frequently employed in both experimental and clinical contexts as indicators of proximal tubular damage (Bonventre, 2009; Clerico et al., 2012).

Although previous reviews have summarized molecular mechanisms of cisplatin-induced nephrotoxicity or described the development of kidney *in vitro* models (Pabla and Dong, 2008; Faria et al., 2019), no systematic evaluation has specifically characterized how biomarker endpoints are selected, biologically categorized, and co-reported in human proximal tubular *in vitro* studies of cisplatin exposure.

Therefore, the present study aimed to systematically characterize the frequency of reported biomarker endpoints, their biological categorization, and their co-reporting patterns in human proximal tubular *in vitro* models of cisplatin-induced injury.

## Methods

### Review design

This systematic review was conducted in accordance with the PRISMA 2020 statement (Page et al., 2021), and the flow diagram was generated using the PRISMA2020 R package (Haddaway et al., 2022). The objective was to synthesize published evidence on biomarkers reported in human proximal tubular *in vitro* models of cisplatin-induced acute kidney injury (AKI) between January 2014 and December 2024. The research question, eligibility criteria, search strategy, biomarker classification framework, and data extraction procedures were defined *a priori* before study selection.

## Protocol and registration

This systematic review was not prospectively registered in PROSPERO or another public registry. The research question, eligibility criteria, search strategy, biomarker classification framework, and data extraction procedures were defined *a priori* before study selection. The review was conducted and reported in accordance with PRISMA 2020 guidance.

### Search strategy

A comprehensive literature search was conducted in PubMed, Scopus, and Web of Science. The search covered studies published between January 2014 and December 2024. The final database search was performed on 26 September 2025 to ensure completeness of indexed records within the predefined publication period. Search terms combined controlled vocabulary and free-text keywords related to cisplatin; acute kidney injury (AKI) or nephrotoxicity; human proximal tubular *in vitro* models (including HK-2, RPTEC/TERT1, primary proximal tubular epithelial cells, induced pluripotent stem cell–derived cells, kidney organoids, and microphysiological systems); and biomarkers. Boolean operators (AND/OR) and database-specific filters were applied as appropriate. Only peer-reviewed articles published in English were eligible for inclusion. The complete database-specific search strings are provided in Supplementary Table S1.

### Eligibility criteria

Studies were considered eligible if they met all of the following criteria: (i) were experimental *in vitro* investigations; (ii) used human-derived proximal tubular epithelial models; (iii) included a clearly defined cisplatin exposure with reported concentration and exposure duration; (iv) incorporated a non-exposed control group; and (v) reported quantifiable molecular or biochemical biomarkers.

Studies were excluded if they: (i) were conducted exclusively in animal models; (ii) used rodent-derived or non–proximal tubular cell lines; (iii) evaluated nephrotoxicity induced by agents other than cisplatin; (iv) did not include a cisplatin-only exposure arm; (v) lacked extractable quantitative biomarker data; or (vi) were review articles, editorials, conference abstracts, or study protocols.

### Study selection

**FIGURE 1:**
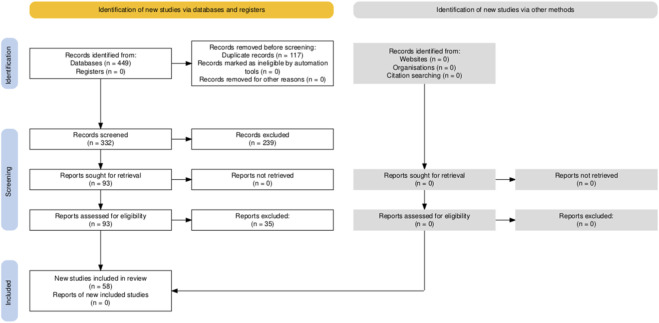
PRISMA 2020 flow diagram of study selection. Records identified through database searching (n = 449). After removal of duplicates (n = 117), 332 records were screened by title and abstract. Of these, 239 were excluded. Ninety-three full-text articles were assessed for eligibility, and 35 were excluded due to insufficient or non-extractable quantitative biomarker data. Fifty-eight studies met all eligibility criteria and were included in the final qualitative synthesis.

A total of 449 records were identified through database searching (PubMed, Scopus, and Web of Science). After removal of 117 duplicate records, 332 unique records were screened by title and abstract. Of these, 239 were excluded for not meeting inclusion criteria. Ninety-three full-text articles were assessed for eligibility. Thirty-five articles were excluded due to insufficient or non-extractable quantitative biomarker data. Fifty-eight studies met all eligibility criteria and were included in the final qualitative synthesis. The study selection process is illustrated in the PRISMA 2020 flow diagram (Figure 1).

### Data extraction

Data were extracted independently by two reviewers using a standardized, pre-piloted Excel matrix. For each included study, the following variables were recorded: (i) type of human proximal tubular cell model; (ii) cisplatin concentration (µM); (iii) exposure duration (hours); (iv) biomarkers evaluated; (v) detection or analytical method; and (vi) primary biological role attributed to each biomarker. To avoid artificial inflation of counts due to multiple experimental conditions within a single study, each biomarker was counted once per article, irrespective of the number of concentrations, exposure times, experimental replicates, or analytical techniques employed.

### Biomarker classification

Biomarkers were classified *a priori* into three predefined biological categories based on their predominant functional role in cisplatin-induced tubular injury: (i) injury biomarkers, reflecting structural or functional damage of proximal tubular epithelial cells (e.g., KIM-1, NGAL); (ii) cell death biomarkers, associated with regulated cell death pathways or terminal membrane integrity loss, including apoptosis, necroptosis, ferroptosis, and related processes (e.g., cleaved caspase-3, RIPK3, MLKL, Annexin V/PI); and (iii) mechanistic biomarkers, reflecting upstream molecular processes involved in nephrotoxicity, such as oxidative stress, mitochondrial dysfunction, inflammatory signaling, or regulated cell death signaling cascades (e.g., ROS, NRF2, IL-6).

The classification framework was defined prior to data extraction to minimize *post hoc* categorization bias. In cases of functional overlap, biomarkers were assigned according to the predominant mechanistic context emphasized in the respective study to ensure consistency in categorization. For category-level analyses, cumulative counts of individual biomarkers were calculated within each predefined category across all included studies. Each biomarker contributed independently to category totals while being counted only once per article to avoid duplication due to multiple experimental conditions within the same study.

### Frequency analysis

A descriptive frequency analysis was conducted to determine the number of included studies reporting each individual biomarker. Biomarkers were ranked according to their frequency of appearance across articles. Complete frequency data are presented in Supplementary Table S3.

### Category distribution analysis

To assess the distribution of biomarker categories across the literature, cumulative counts of individual biomarkers classified within each predefined biological category were calculated. Category totals therefore represent the total number of biomarker reports within each category across all included studies, with each biomarker counted once per article. Category-level counts are provided in Supplementary Table S4.

### Co-occurrence analysis

To examine structured patterns of biomarker usage across studies, a descriptive co-occurrence network analysis was conducted focusing on the 12 most frequently reported biomarkers. A binary presence–absence matrix was constructed to indicate whether each biomarker was reported in each individual study. Based on this matrix, pairwise co-occurrence counts were calculated, representing the number of independent studies in which two biomarkers were reported together within the same article.

The selection of the top 12 biomarkers was based on frequency ranking, allowing the analysis to concentrate on the most consistently reported endpoints while avoiding excessive network complexity. Co-occurrence relationships were visualized as an undirected network, where node size reflects reporting frequency and edge thickness corresponds to the number of studies in which two biomarkers co-occurred. Only pairwise relationships reported in at least three independent studies were retained. This threshold was selected to reduce random co-reporting patterns and enhance interpretability of consistent reporting structures. This analysis was descriptive in nature and did not involve statistical inference testing. Complete pairwise co-occurrence data are provided in Supplementary Table S5.

### Statistical approach

Due to substantial heterogeneity across studies in terms of cell models, cisplatin concentrations, exposure durations, biomarker panels, and reporting formats, a formal quantitative meta-analysis was not undertaken. Instead, a structured descriptive synthesis complemented by matrix-based co-occurrence analysis was performed to characterize patterns of biomarker utilization.

No formal risk-of-bias assessment was conducted because the included studies comprised heterogeneous mechanistic *in vitro* experimental designs for which no validated appraisal tool currently exists. The objective of this review was descriptive characterization of biomarker reporting patterns rather than estimation of treatment effects.

### Data visualization

All statistical and graphical analyses were conducted in R (version 4.1.3). Frequency ranking and category distribution plots were generated using the ggplot2 package. The co-occurrence network was constructed from a binary presence–absence matrix and visualized using the igraph package with a force-directed layout algorithm. Network visualization parameters were standardized, and all figures were exported in high-resolution formats suitable for journal submission.

## Results

A total of 58 studies were included in the final analysis of biomarkers reported in human *in vitro* models of cisplatin-induced acute kidney injury (AKI).

The study-level evidence base used for the descriptive synthesis comprised experimental reports evaluating cisplatin-induced renal tubular injury, nephrotoxicity biomarkers, regulated cell death, oxidative stress, mitochondrial dysfunction, inflammatory signaling, transcriptomic responses, nephroprotective interventions, epithelial–mesenchymal transition, fibrosis-related responses, and advanced renal *in vitro* platforms. These studies included human proximal tubular models such as HK-2 cells, RPTEC/TERT1 cells, primary or stem cell–derived renal epithelial cells, kidney organoids, tubuloids, tubule arrays, and microphysiological systems, as well as mixed *in vivo*/*in vitro* studies with extractable human tubular cell data, as detailed in Supplementary Table S2.

Included studies using conventional or advanced human renal *in vitro* platforms included two-dimensional proximal tubular cell models, immortalized proximal tubular epithelial cells, stem cell-derived renal cells, kidney organoids, tubuloids, tubule arrays, and microphysiological systems (Adler et al., 2016; Aschauer et al., 2015; Digby et al., 2020; Guo et al., 2020; Kim et al., 2014; Li et al., 2014; Maass et al., 2019; Mody et al., 2024; Mossoba et al., 2016; Qiu et al., 2018; Qiu et al., 2020; Sallustio et al., 2017; Sinaeve et al., 2022; Subramanian et al., 2018; Susa et al., 2023; Tang et al., 2022; Vormann et al., 2018; Wiraja et al., 2021).

Additional extracted studies addressed cisplatin-induced tubular injury, nephroprotective interventions, inflammatory signaling, oxidative stress, apoptosis, necroptosis, pyroptosis, ferroptosis, mitochondrial dysfunction, autophagy, epithelial–mesenchymal transition, fibrosis-related responses, transcriptomic signatures, and injury-associated readouts such as KIM-1, NGAL, IL-18, NAG, BUN, creatinine, cytokines, oxidative stress markers, and regulated cell death markers (Balkrishna et al., 2024; Becerir et al., 2021; Cai et al., 2024; Chen et al., 2020; Cui et al., 2022; Deng et al., 2023; Gao et al., 2024; Hu et al., 2020; Jones et al., 2023; Luo et al., 2023; Meng et al., 2017; Shen et al., 2021; Song et al., 2022; Soni et al., 2018; Soni et al., 2019; Tristão et al., 2016; Wang et al., 2022; Wang et al., 2025; Wei et al., 2022; Wu et al., 2020; Xiao et al., 2022; Xie et al., 2022; Xie et al., 2023; Xu et al., 2021; Xu et al., 2023; Yan et al., 2017; Yang Q. et al., 2020; Yang Q. et al., 2021; Yang et al., 2018; Yang et al., 2020a; Yang et al., 2020b; Yang Y. Y. et al., 2021; Yu et al., 2022; Zang et al., 2019; Zhang J. et al., 2020; Zhang S. et al., 2020).

### Frequency ranking of reported biomarkers

**FIGURE 2:**
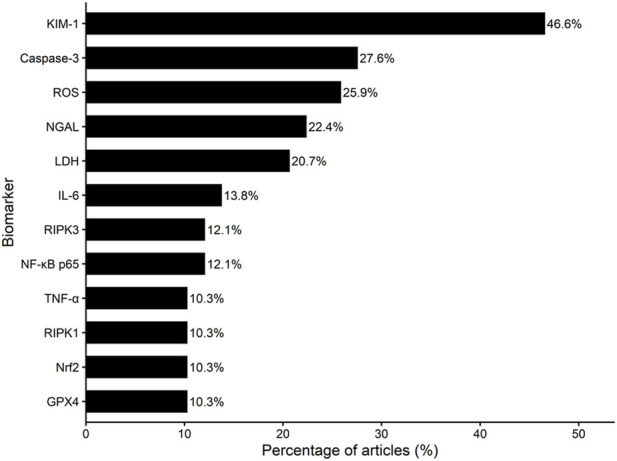
Frequency ranking of biomarkers reported in cisplatin-induced acute kidney injury studies using human proximal tubular *in vitro* models.

The frequency analysis revealed a structured distribution pattern of biomarker usage across included studies (Figure 2).

Kidney injury molecule-1 (KIM-1) was the most frequently reported biomarker, appearing in 27 of 58 studies (46.6%). Caspase-3 represented the second most frequently assessed endpoint, followed closely by reactive oxygen species (ROS). Neutrophil gelatinase-associated lipocalin (NGAL) and lactate dehydrogenase (LDH) followed with lower but comparable reporting frequencies.

Markers of apoptosis, including cleaved caspase-3, caspase-3, and Annexin V/PI, were reported with moderate frequency. Necroptosis-associated markers (RIPK1, RIPK3, and phosphorylated MLKL) and the ferroptosis-related enzyme GPX4 were less frequently reported but were observed across multiple independent studies.

Each biomarker was counted once per study, irrespective of the number of experimental conditions or analytical techniques employed. Complete frequency values are provided in Supplementary Table S3. These frequency data served as the basis for the subsequent co-occurrence network analysis.

Bars represent the number of independent studies reporting each biomarker. Each biomarker was counted once per study, irrespective of the number of experimental conditions or analytical techniques employed. Complete frequency data are provided in Supplementary Table S3.

### Distribution of biomarker categories

**FIGURE 3:**
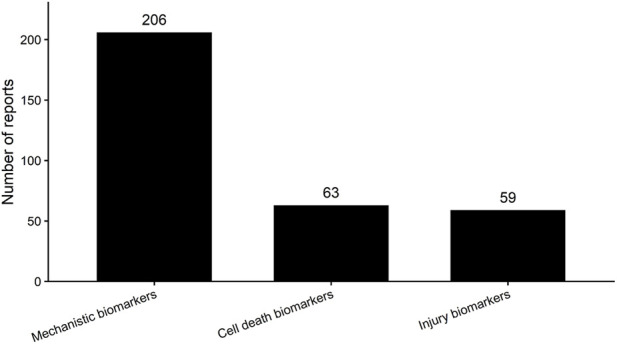
Distribution of biomarker categories in cisplatin-induced acute kidney injury studies using human proximal tubular *in vitro* models. Category totals represent cumulative biomarker reporting frequencies across included studies, with each biomarker counted once per article. Mechanistic biomarkers include oxidative stress, mitochondrial dysfunction, inflammatory signaling, and regulated cell death–related endpoints. Detailed counts by biomarker and category are provided in Supplementary Table S4.

When biomarkers were grouped according to predefined biological roles, mechanistic endpoints accounted for the highest cumulative number of reports (Figure 3).

When cumulative biomarker occurrences were analyzed (each biomarker counted once per study), mechanistic biomarkers accounted for 206 reports, exceeding cell death biomarkers (n = 63) and structural injury biomarkers (n = 59).

Mechanistic biomarkers included oxidative stress indicators, mitochondrial dysfunction markers, inflammatory mediators, and regulated cell death–related signaling components. Cell death biomarkers comprised endpoints related to apoptosis, necroptosis, ferroptosis, and membrane integrity disruption. Injury biomarkers reflected proximal tubular structural damage markers.

Category totals reflect cumulative reporting frequencies rather than the number of independent studies. Detailed counts by biomarker and category are provided in Supplementary Table S4.

### Co-occurrence network analysis

**FIGURE 4:**
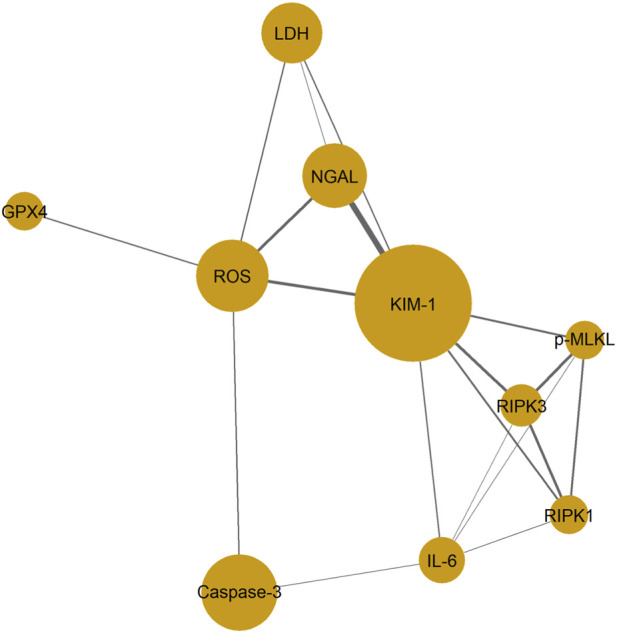
Descriptive co-occurrence network of frequently reported *in vitro* biomarkers in cisplatin-induced nephrotoxicity (threshold ≥3 studies).

To explore patterns of combined biomarker usage, a descriptive co-occurrence network analysis was performed including the 12 most frequently reported biomarkers (Figure 4). Edges represent biomarker pairs co-reported in at least three independent studies (threshold ≥3).

The resulting network showed KIM-1 co-reported with oxidative stress markers (ROS), structural injury markers (NGAL, LDH), apoptosis-related endpoints (caspase-3), inflammatory mediators (IL-6), and necroptosis-associated markers (RIPK1, RIPK3, and p-MLKL). Prominent co-reporting relationships were observed between KIM-1 and NGAL, and between KIM-1 and ROS.

ROS displayed multiple co-reporting relationships linking structural injury markers (NGAL, LDH) with regulated cell death markers (caspase-3 and GPX4).

Recurrent co-reporting among RIPK1, RIPK3, and p-MLKL was observed within the necroptosis-associated group. These markers were also co-reported with KIM-1 and IL-6 in multiple studies.

Apoptosis-related biomarkers appeared broadly distributed within the network and were co-reported with structural and mechanistic markers across studies.

Overall, the network demonstrates recurrent multi-endpoint reporting patterns rather than isolated biomarker usage. The complete pairwise co-occurrence matrix underlying this network is provided in Supplementary Table S5.

Nodes represent the 12 most frequently reported biomarkers across included studies. Node size is proportional to the number of independent studies reporting each biomarker. Edges represent biomarker pairs co-reported within the same study and are shown only when reported together in at least three independent studies (threshold ≥3). Edge thickness reflects the number of studies in which the biomarker pair co-occurred. The network is undirected and was constructed from a binary presence–absence matrix. This visualization is descriptive and does not involve quantitative effect size pooling or formal graph-theoretical community detection.

#### Experimental characteristics of studies reporting KIM-1

Among all included biomarkers, KIM-1 was the most frequently reported structural injury marker.

Most investigations employed the HK-2 human proximal tubular cell line, followed by RPTEC/TERT1 cells and, less frequently, kidney organoid models. Cisplatin concentrations commonly ranged between 20 and 40 μM, with 20 µM representing the most frequently used exposure level. Higher concentrations were observed in selected exploratory and transcriptomic studies. Exposure duration was predominantly 24 h, followed by 48-h protocols.

KIM-1 quantification was primarily performed using quantitative PCR and Western blot analysis, whereas organoid-based systems more frequently incorporated immunostaining approaches. In most studies, intracellular expression was measured rather than extracellular release.

Variability was observed in normalization strategies and reporting units, particularly in qPCR-based quantification (ΔCt versus 2^-ΔΔCt^ methods) and densitometric analyses.

Detailed experimental characteristics, including cell model, cisplatin concentration, exposure duration, and analytical method, are provided in Supplementary Table S2.

## Discussion

### Principal findings

This systematic review provides a structured synthesis of biomarkers reported in human proximal tubular *in vitro* models of cisplatin-induced acute kidney injury (AKI) between 2014 and 2024. Three principal observations emerge from the analysis.

First, KIM-1 was the most consistently reported biomarker, appearing in nearly half of included studies. Its frequent use across heterogeneous experimental platforms reinforces its established role as a structural indicator of proximal tubular injury in cisplatin-exposed human renal epithelial models.

Second, mechanistic biomarkers clearly predominated when cumulative reporting frequencies were analyzed. Oxidative stress indicators, mitochondrial dysfunction markers, and intracellular signaling mediators accounted for the highest total number of reports, substantially exceeding both cell death and structural injury biomarkers. This distribution indicates that contemporary *in vitro* studies primarily emphasize pathway-level mechanistic investigation.

Third, co-occurrence network analysis demonstrated reproducible integration patterns of biomarker usage. Rather than appearing as isolated endpoints, frequently reported biomarkers tended to be assessed in combination within multi-endpoint experimental panels.

### Predominance of mechanistic endpoints

The marked predominance of mechanistic biomarkers suggests that *in vitro* cisplatin nephrotoxicity research is strongly oriented toward elucidating molecular pathways underlying tubular injury. Oxidative stress, mitochondrial dysfunction, and intracellular signaling endpoints were consistently incorporated, often alongside structural and regulated cell death markers.

Although structural injury biomarkers such as KIM-1 were widely used, their cumulative reporting frequency was lower than that of mechanistic endpoints. Similarly, cell death biomarkers—particularly apoptosis-related markers—were moderately represented but did not exceed mechanistic categories.

This distribution reflects the exploratory and hypothesis-driven nature of *in vitro* experimental designs, where mechanistic pathway interrogation often takes precedence over standardized injury validation frameworks.

### Structured co-occurrence patterns

The co-occurrence network analysis further supports the presence of structured biomarker integration. KIM-1 appeared as the most highly connected node within the network, linking structural injury assessment with oxidative stress (ROS), regulated cell death markers (Caspase-3, RIPK1, RIPK3, p-MLKL), and inflammatory mediators (IL-6). This recurrent integration suggests that structural injury endpoints are frequently assessed in parallel with mechanistic and regulated cell death pathways.

A visually identifiable necroptosis-associated grouping composed of RIPK1, RIPK3, and p-MLKL was observed, reflecting their recurrent co-reporting within studies investigating regulated necrotic pathways. Apoptosis-related markers appeared broadly integrated within the network rather than forming isolated pathway-restricted panels.

Importantly, this analysis is descriptive and does not imply causal or hierarchical pathway relationships. Instead, it illustrates how investigators assemble biomarker combinations within experimental designs. The recurrent integration of injury, oxidative stress, and regulated cell death endpoints suggests pathway-oriented panel construction rather than convergence toward standardized cross-study biomarker frameworks.

## Model heterogeneity and translational implications

Substantial heterogeneity across cell models, cisplatin concentrations, exposure durations, and analytical techniques remains a defining feature of the field. While HK-2 cells were frequently used, alternative platforms such as RPTEC/TERT1 cells and kidney organoids introduce variability in transporter expression, metabolic competence, and phenotypic stability, potentially influencing biomarker responsiveness.

Variability in detection methodologies and reporting formats further limits direct cross-study comparability. Notably, intracellular expression was more commonly measured than extracellular release, potentially constraining translational extrapolation to clinical urinary biomarker settings.

Nevertheless, the consistent prominence and high connectivity of KIM-1 across heterogeneous experimental contexts support its potential utility as a translational bridge between mechanistic *in vitro* systems and clinically established urinary injury markers.

### Practical biomarker prioritization and standardization

From a practical and translational perspective, the most promising biomarkers for future standardization are those that combine frequent reporting, biological relevance to proximal tubular injury, and mechanistic interpretability. KIM-1 appears particularly relevant because it was the most consistently reported structural injury biomarker and showed recurrent co-reporting with oxidative stress, inflammatory, and regulated cell death endpoints. NGAL/LCN2 may also be useful as a complementary injury and stress-associated biomarker, although its interpretation may be influenced by broader cellular stress responses.

Clusterin, N-acetyl-β-D-glucosaminidase (NAG), and osteopontin (OPN) may provide additional value as complementary indicators of tubular stress, lysosomal injury, and tissue remodeling responses, respectively.

Mechanistic endpoints such as ROS generation, oxidative stress markers, mitochondrial dysfunction indicators, and caspase-3 activity provide important information on the biological pathways involved in cisplatin-induced tubular injury. However, these endpoints should not be interpreted as direct substitutes for structural injury biomarkers. Instead, future *in vitro* nephrotoxicity studies may benefit from standardized biomarker panels that combine structural injury markers, cellular stress indicators, oxidative damage endpoints, mitochondrial dysfunction markers, and regulated cell death biomarkers.

At present, standardized cutoff values, universal threshold definitions, or reference ranges are not established for major biomarkers such as KIM-1, NGAL/LCN2, ROS, or caspase-3 in human proximal tubular *in vitro* models exposed to cisplatin. Biomarker responses are highly dependent on the cell model, cisplatin concentration, exposure duration, assay platform, normalization strategy, and reporting format. Therefore, future studies should report biomarker changes relative to matched untreated controls, provide dose–response and time-course information, define normalization procedures explicitly, and predefine the criteria used to classify a biomarker response as biologically meaningful.

## Strengths and limitations

This review applies a systematic PRISMA-guided methodology, focuses exclusively on human proximal tubular *in vitro* models, and integrates frequency ranking, categorical distribution analysis, and descriptive co-occurrence mapping to characterize biomarker reporting architecture.

However, several limitations warrant consideration. This systematic review was not prospectively registered in PROSPERO or another public registry, which limits external verification of *a priori* methodological decisions and means that the possibility of methodological deviations or selective reporting cannot be completely excluded. In addition, the lack of standardized cutoff values or reference ranges for major *in vitro* biomarkers limited the possibility of comparing threshold-based biomarker responses across studies. The analysis was restricted to English-language publications. Heterogeneity in experimental design precluded quantitative meta-analysis. Co-occurrence patterns reflect reporting behavior rather than mechanistic dependency, and reporting frequency does not necessarily equate to diagnostic performance, predictive validity, or biological hierarchy.

## Future directions

Future research would benefit from greater methodological harmonization, including standardized cisplatin exposure windows, consistent reporting units, explicit normalization strategies, and predefined criteria for interpreting biomarker responses. Inclusion of established structural injury markers—such as KIM-1 and NGAL/LCN2—alongside mechanistic endpoints such as ROS generation, mitochondrial dysfunction, inflammatory signaling, and caspase-3 activity may improve cross-study comparability and translational relevance.

Comparative validation of biomarker panels across multiple human proximal tubular platforms is warranted. Integration of multi-marker panels combining structural injury, oxidative stress, inflammation, and regulated cell death pathways may provide a more comprehensive assessment of nephrotoxicity.

Such harmonization efforts would enhance reproducibility, strengthen translational alignment, and facilitate development of consensus biomarker frameworks in vitro nephrotoxicity research. Development of consensus reporting frameworks and minimal experimental reporting standards may further enhance comparability and accelerate translational validation.

## Conclusion

Human proximal tubular *in vitro* models of cisplatin-induced acute kidney injury (AKI) demonstrate structured patterns of biomarker utilization characterized by a clear predominance of mechanistic endpoints, accompanied by consistent inclusion of structural injury and regulated cell death markers.

KIM-1 remains the most consistently reported structural biomarker and exhibited the highest number of co-reporting relationships within the descriptive co-occurrence network, frequently appearing alongside oxidative stress, inflammatory, and regulated cell death endpoints.

Recurrent integration of structural injury, oxidative stress, and regulated cell death markers suggests hypothesis-driven multi-endpoint panel construction rather than convergence toward standardized cross-study biomarker frameworks. While this approach advances mechanistic understanding, substantial heterogeneity in exposure conditions, analytical methods, and reporting strategies limits direct cross-study comparability.

Greater methodological harmonization, including standardized exposure windows, consistent normalization strategies, and structured reporting frameworks, may enhance reproducibility, improve translational alignment, and facilitate development of consensus biomarker panels for *in vitro* nephrotoxicity assessment. The absence of standardized *in vitro* cutoff values for commonly used biomarkers further supports the need for relative, model-specific, and assay-specific reporting frameworks.

## Data Availability

The original contributions presented in the study are included in the article/Supplementary Material, further inquiries can be directed to the corresponding author.

## Supplementary material

SUPPLEMENTARY TABLE S1 Database-specific search strategies.

SUPPLEMENTARY TABLE S2 Characteristics of included studies and extracted biomarker information.

SUPPLEMENTARY TABLE S3 Frequency ranking of reported biomarkers.

SUPPLEMENTARY TABLE S4 Biomarker category distribution.

SUPPLEMENTARY TABLE S5 Pairwise biomarker co-occurrence matrix.
